# Folic Acid Transport to the Human Fetus Is Decreased in Pregnancies with Chronic Alcohol Exposure

**DOI:** 10.1371/journal.pone.0038057

**Published:** 2012-05-29

**Authors:** Janine R. Hutson, Brenda Stade, Denis C. Lehotay, Christine P. Collier, Bhushan M. Kapur

**Affiliations:** 1 Division of Clinical Pharmacology and Toxicology, Hospital for Sick Children, Toronto, Ontario, Canada; 2 Institute of Medical Science, University of Toronto, Toronto, Ontario, Canada; 3 Department of Paediatrics, Keenan Research Centre, St. Michael's Hospital, Toronto, Ontario, Canada; 4 College of Medicine, University of Saskatchewan, Regina, Saskatchewan, Canada; 5 Department of Pathology and Molecular Medicine, Queen's University, Kingston, Eastern Ontario, Canada; 6 Kingston General Hospital, Kingston, Eastern Ontario, Canada; 7 Department of Clinical Pathology, Sunnybrook Health Sciences Centre, Toronto, Ontario, Canada; 8 Department of Laboratory Medicine and Pathobiology, Faculty of Medicine, University of Toronto, Ontario, Canada; Medical Faculty, Otto-von-Guericke University Magdeburg, Germany

## Abstract

**Background:**

During pregnancy, the demand for folic acid increases since the fetus requires this nutrient for its rapid growth and cell proliferation. The placenta concentrates folic acid into the fetal circulation; as a result the fetal levels are 2 to 4 times higher than the maternal level. Animal and *in vitro* studies have suggested that alcohol may impair transport of folic acid across the placenta by decreasing expression of transport proteins. We aim to determine if folate transfer to the fetus is altered in human pregnancies with chronic alcohol consumption.

**Methodology/Principal Findings:**

Serum folate was measured in maternal blood and umbilical cord blood at the time of delivery in pregnancies with chronic and heavy alcohol exposure (n = 23) and in non-drinking controls (n = 24). In the alcohol-exposed pairs, the fetal∶maternal serum folate ratio was ≤1.0 in over half (n = 14), whereas all but one of the controls were >1.0. Mean folate in cord samples was lower in the alcohol-exposed group than in the controls (33.15±19.89 vs 45.91±20.73, p = 0.04).

**Conclusions/Significance:**

Our results demonstrate that chronic and heavy alcohol use in pregnancy impairs folate transport to the fetus. Altered folate concentrations within the placenta and in the fetus may in part contribute to the deficits observed in the fetal alcohol spectrum disorders.

## Introduction

**Figure 1:**
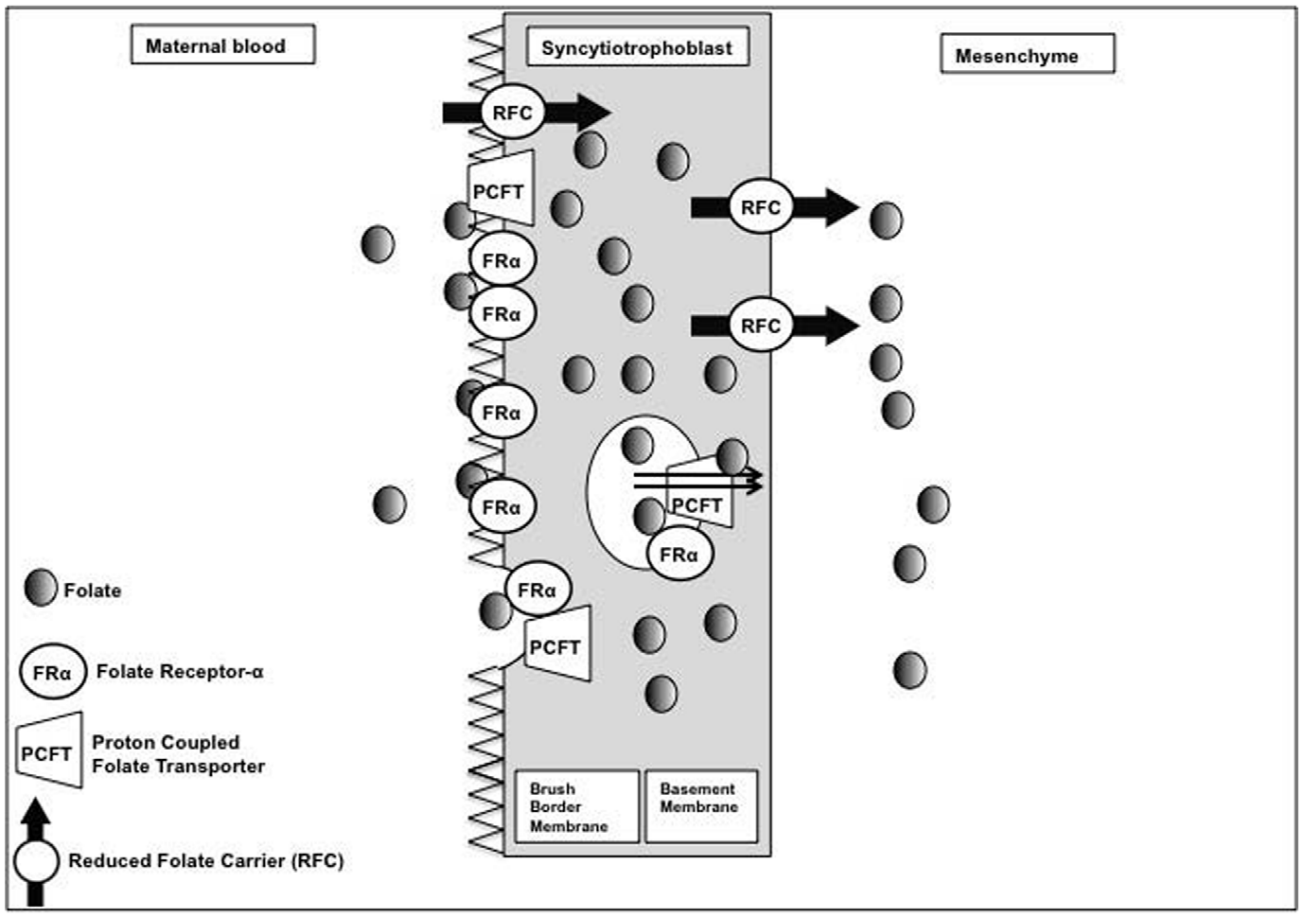
The transport proteins present on the syncytiotrophoblast that are involved in the transfer of folate to the fetal circulation.

During pregnancy, the demand for folic acid increases since this nutrient is critically important for DNA synthesis and cell proliferation. During pregnancy, the placenta concentrates folic acid into the fetal circulation and as a result fetal levels are 2- to 4-fold higher than maternal [1]–[9]. The fetal requirement for folate during pregnancy is paramount such that cord folate status is maintained even when maternal status is low [10]. Transport of folates across the placenta is mediated by placental folate receptors (PFRs) [11], namely folate receptor-α (FR-α) at the microvillous membrane of the syncytiotrophoblast [12], [13]. Folate in the maternal circulation binds to FR-α, where it binds with high affinity and is internalized through receptor-mediated endocytosis [14] (Figure 1). Other transporters including the reduced folate carrier (RFC) and the proton-coupled folate transporter (PCFT) are also important for the placental uptake of folate [15], [16]. This results in a high intervillous blood concentration of folate within the placenta where it can be transported to the fetus by passive diffusion and by the RFC [11], [12]. This mechanism of transport is established early in pregnancy (within the first trimester [13]) as folate is vital to the proper development of the fetus.

Animal and *in vitro* studies have suggested that alcohol may impair transport of folic acid across the placenta by decreasing expression of folate transport proteins [14], [17], [18]. Indeed, there are similarities between deficits observed in the fetal alcohol spectrum disorders (FASD) and folate status during pregnancy. These similarities include common physical malformations such as neural tube defects, congenital heart defects, and limb defects [19]–[21]. Furthermore, lower folate status has been associated with hyperactivity, peer problems, and lower cognitive function [22], [23], which are all neuro-developmental consequences of alcohol exposure during pregnancy [20].

Folic acid is also important as an antioxidant during pregnancy. Alcohol consumption during pregnancy creates oxidative stress to both the placenta and fetus and this stress can be mitigated by folic acid [24], [25]. Formic acid, the toxic metabolite of methanol, has been reported in the sera of alcohol abusing patients and has also recently been detected in umbilical cord blood from pregnancies with heavy amounts of alcohol consumption [26]. Formic acid can lead to neurotoxicity and oxidative stress [27]. Folic acid is required for the detoxification of formic acid. Folic acid is critical to the rate of detoxification of formic acid [28]. Furthermore, in folate deficient animals, the adverse effects to the fetus after alcohol exposure are more severe compared to controls [29], [30]. Taken together, proper placental transfer of folic acid is critical to proper fetal development and can influence the fetal effects of alcohol.

To our knowledge, there are no studies that determine fetal folate levels from pregnancies affected by heavy alcohol use. We hypothesize that folate transfer to the fetus is impaired in alcohol-exposed pregnancies and that this may, in part, be responsible for the deficits associated with the fetal alcohol spectrum disorders (FASDs).

## Methods

All procedures and protocols received prior approval from the Institutional Research Ethics Board. The most severe diagnosis under the FASDs is fetal alcohol syndrome (FAS). Children with FAS are usually born to mothers who consume large amounts of alcohol in the pregnancy and for a long duration. However, there is large variation in the deficits produced with alcohol consumption and likely results from differences in amount consumed, drinking patterns, genetics, and nutrition. Only women consuming heavy amounts throughout the pregnancy were included in this study and are thus the women most at risk of having a child affected by FAS. This avoided any variability that may result from differences in amount of alcohol consumed and timing of exposure. Healthy women with no known illicit drug or alcohol use during pregnancy were also recruited to be representative of the general population to serve as our comparison group. As part of this study, the infants were offered follow up care and diagnostic testing for the FASDs.

Written informed consent was obtained from the alcohol abusing mothers included in this study. Maternal and cord blood samples were collected at the time of delivery. These women were identified at the time of delivery and questionnaires regarding drug use were administered after delivery. For controls maternal and cord blood samples were collected as part of routine clinical workup of women delivering at the hospital. Plasma folic acid was measured in our routine clinical laboratory by chemiluminescent immunoassay using the Beckman Coulter UniCel DxI 800 Access® Immunoassay System (Beckman Coulter Canada, Inc., Mississauga, Ont. Canada). Manufacturer recommended assay protocol was used. Besides folic acid results no other information on these women was available to us.

Normally distributed data were compared as follows: maternal and fetal folate levels were compared using a paired t-test; folate levels in the control group compared to the alcohol group were compared using an independent t-test. Pearson correlation was used to compare maternal and fetal folate levels. Fetal to Maternal folate ratios were compared using the Mann Whitney U Test because they were not normally distributed. Significance was obtained if p<0.05 and all performed tests were two-tailed (where applicable).

## Results and Discussion

Twenty-three women consuming heavy amounts throughout the pregnancy were included in this study. Twenty-four women were recruited from the general population to serve as the comparison group with no known illicit drug or alcohol use during pregnancy. Maternal demographics and fetal information for the women drinking alcohol during pregnancy is given in Table 1. Maternal age for this group ranged from 16 to 44 years. All women reported regular alcohol consumption throughout the pregnancy and were considered to be dependent users. The women were not recruited until later in their pregnancy and self-reported alcohol consumption ranged from daily use to >8 drinks per week. The women included in this cohort were being followed by an FASD clinic and were therefore considered at high risk of having an affected child because of their alcohol consumption. In addition to alcohol, fourteen women reported occasional to frequent cocaine use, eight reported marijuana use, and two reported opiate use during the pregnancy. Cigarette use was also common to this population.

**Table 1:** Demographic information on alcohol-using women included in this study and fetal parameters. (n = 23).

	Mean (SD)
Maternal age	29.2 (6.9) years
Gravidity	4.8 (3.4)
Parity	2.4 (1.9)
Gestational age	36.6 (1.6) weeks
Length	42.3 (3.2) cm
Head Circumference	32.3 (1.4) cm
Birth weight	2830 (421) g

**Figure 2:**
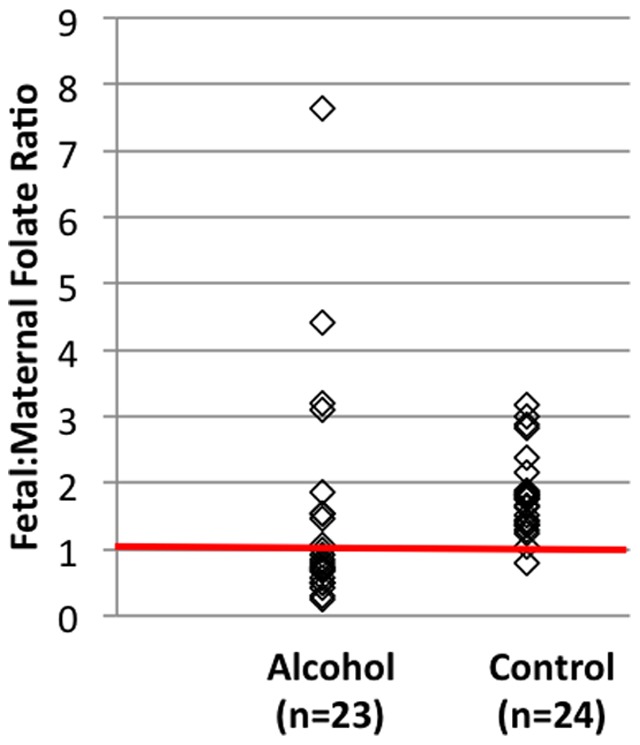
Scatter-plot of the fetal to maternal (F∶M) folate ratios as measured in cord blood and maternal blood, respectively, at the time of delivery.

**Figure 3:**
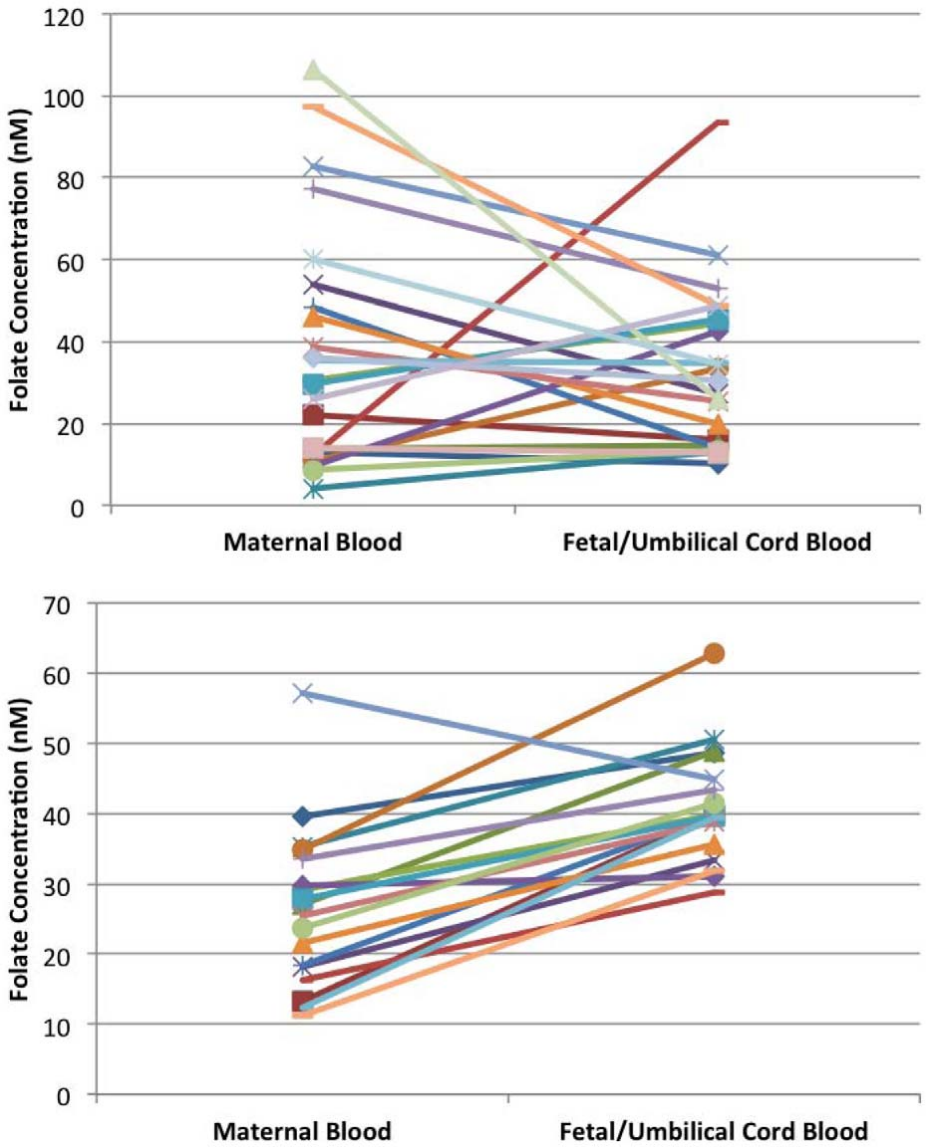
Corresponding maternal and fetal folate concentrations at the time of delivery in pregnancies with (A) heavy alcohol exposure and (B) in controls.

In order to determine whether the placenta was concentrating folate to the fetal circulation, we calculated the fetal to maternal (F∶M) folate ratio using the serum folate measurements from the mother-cord pairs. The F∶M folate ratio was ≤1.0 in over half (n = 14) of the alcohol-exposed pairs, whereas all but one of the controls were >1.0 (Figure 2). The F∶M folate ratios in the alcohol group were significantly lower than the control group (p = 0.014). Also, there was large variability observed in the F∶M folate ratios in the alcohol group (range 0.24 to 7.65) and not in the control group (range 0.79 to 3.18), suggesting that the tight regulation of folate transport to the fetus is deregulated (Figure 3).

**Figure 4:**
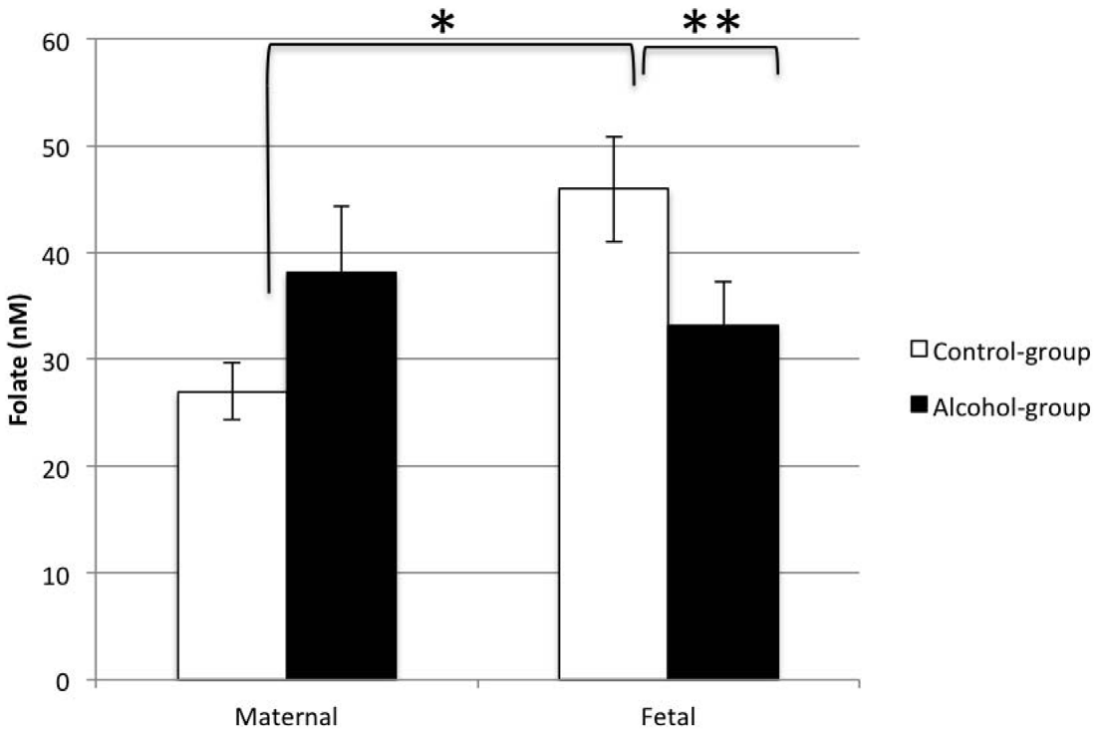
Mean (± SEM) cord and maternal plasma folate concentrations at the time of delivery in alcohol-abusing women and controls. *p<0.05 for a two-tailed paired t-test. **p<0.05 for a two-tailed independent t-test.

As expected, the fetal folate levels in the control group were significantly higher than maternal in controls (45.91 nM±20.73 nM vs 26.99 nM±11.18 nM, p<0.0001) (Figure 4). This finding has been reported in all studies to date comparing maternal and fetal folate levels [1]–[9]. However, this finding was not observed in the alcohol group and there was a trend for lower fetal levels compared to maternal (33.15 nM±19.89 nM vs 38.13 nM±29.55 nM, respectively, p = 0.461). Furthermore, there was no correlation between the maternal and cord folate levels in the alcohol group (R^2^ = 0.051, p = 0.226), yet there was the expected correlation in the control group (R^2^ = 0.550, p = 0.018). Together, these data support folate transport being impaired in pregnancies affected by chronic and heavy alcohol use. Because only half of mother-fetal pairs had a F∶M folate ratio of less than one, this suggests variability in the effect. This could result from differences in drinking patterns, genetic susceptibility, or other drug use in addition to alcohol (to be discussed below).

After observing an impaired ability to concentrate folic acid to the fetus, we next wanted to see if the actual folate concentration in the fetal circulation was lower in the alcohol-exposed group. Indeed, folate levels were significantly lower in cord blood in alcohol-exposed fetuses compared to controls (p = 0.04). Interestingly, there was no difference in folate levels between the alcohol-using and control women. There was a trend for increased folate levels in the alcohol group. This was not expected since folate deficiency is common with chronic alcohol consumption [31]. Two studies however, have reported higher folate levels in alcohol-using pregnant women compared to controls and may reflect women drinking beer, which contains folates [32], [33]. Despite having higher maternal folate levels, fetal folate levels were still lower in the alcohol group – emphasizing that alcohol likely deregulates placental folate transport.

It has been suggested that alcohol consumption during pregnancy may alter the placental transport of folate in animal and *in vitro* studies; however, no conclusion has been reached. *In vitro* studies using both a choriocarcinoma cell line (BeWo) and primary trophoblast cells from human term placentas demonstrated decreased folic acid uptake after chronic exposure to ethanol [14], [18]. Furthermore, after chronic exposure in rats, there was a decrease in folic acid binding [17]. Conversely, other studies have shown that alcohol may not alter folate transport. Dual-perfusion of a single lobule from a term human placenta did not demonstrate altered folate binding or transfer after treatment with ethanol [11]. Similarly, a single ethanol exposure to rats during pregnancy did not alter transport of folate [29]. The different findings likely result from the type of exposure since only those studies where there was chronic alcohol exposure showed a decreased effect. Our study population in the alcohol group was consuming alcohol continuously during pregnancy and is in concordance with the chronic animal and in vitro studies.

Decreased folate transfer may be a result of altered expression of the folate binding and transport proteins, including FR-α, RFC, and PCFT. After chronic alcohol exposure *in vitro*, primary trophoblasts and BeWo cells had reduced mRNA expression of RFC and FR-α respectively [14], [18]. However, there are no data available looking at mRNA, protein, or folic acid uptake in human placenta from a population similar to that in our study. Future studies should investigate this mechanism using human placentas from alcohol-exposed pregnancies. Since this population is difficult and sensitive to recruit and has many co-morbidities, animal studies may be crucial to a full mechanistic understanding.

A role for impaired folic acid transport in the development of FASDs is further supported by animal studies. After alcohol exposure during pregnancy in mice and rats, more adverse fetal effects were observed in those born to mothers receiving a folate free diet [30], [34]. Furthermore, several recent animal studies have reported that high dose folic acid can mitigate the adverse fetal effects induced by alcohol, especially measures of oxidative stress [25], [35]–[38]. A recent study has demonstrated that administration of folic acid by injection decreases fetal resorption and malformations in mice consuming ethanol in pregnancy [39]. These findings suggests that it may be possible to overcome the decreased placental transfer observed in this study if maternal levels are high enough.

The fetotoxic effects resulting from folate deficiency may result from different possible pathways. Folic acid is critically important for DNA synthesis and cell proliferation as well as in cellular protection through its antioxidant properties [40]. Folate deficiency within the placenta has also been shown to alter placental DNA methylation [41]. Fetal growth and development are closely linked to DNA methylation [41]. Disruption in placental DNA methylation may have a role in the Developmental Origins of Adult Health and Disease hypothesis and alterations in the placenta have already been associated with the future risk of cardiovascular disease and cancer [42].

An additional complication that may arise from folate deficiency is related to formic acid, the toxic metabolite of methanol. Formic acid has recently been detected in maternal and cord blood in pregnancies with chronic ethanol exposure [26]. This formic acid is endogenously produced from methanol naturally found in alcoholic beverages as well as methanol produced as a by-product in the pituitary [43]. Formic acid requires folic acid for detoxification. A recent study by our group observed a negative correlation between formic acid in cord blood and cognitive function (r = −0.6154, p = 0.025, n = 12 at 12 months and r = −0.6241, p = 0.023, n = 13 at 18 months) using Bayley scores [26]. Formic acid has been shown to cause neuronal cell death in the rat hippocampal explants and this could be mitigated by the folic acid [27]. Thus, lower levels of folate may leave the fetus more vulnerable to the potential toxic effects of formic acid.

The women included in our study in the alcohol group also reported use of other drugs of abuse, including cocaine, tetrahydrocannabinol (THC in marijuana), and cigarettes. The influence of these drugs on folate transfer cannot be ruled out. However, *in vitro* studies do not support a role for cocaine in altering folate transport. Studies using primary trophoblasts showed that acute or chronic exposure to cocaine did not alter folate uptake [14]. However, chronic exposure (but not acute) to THC, did decrease folate uptake in primary trophoblasts [14]. Furthermore, amphetamine, and ectasy (MDMA) decreased folate uptake after both acute and chronic exposure. No women in our study reported use of amphetamine or ecstasy, however, self-report may not be 100% accurate due to the stigma associated with illicit drug use. Also, it is unknown whether the reported occasional use of THC by the three women in this study would be enough to cause an effect on folate transport since chronic exposure was needed in the in vitro studies to have an effect.

There are currently no *in vivo* data available that relate folate transport to drugs of abuse; however, there are studies evaluating folate transport in pregnancy to cigarette smoking in pregnancy. A small decrease in cord folate levels was determined in infants born to smoking mothers compared to non-smoking mothers [8]. However, the fetal folate levels remained higher than maternal and the positive linear relationship was maintained in the smoking group. The decrease in fetal folate levels and transfer was more profound in our study population; thus the observed effect in our study is a result of more than simply maternal cigarette use. An interaction or additive effect between smoking, alcohol, and other drugs of abuse may be possible and should be further investigated.

To our knowledge, this is the first study to show that folic acid transport to the fetus is compromised in pregnancies affected by heavy and chronic alcohol exposure. Decreased levels of folate available to the fetus as well as bound to the placenta itself may in part be responsible for the deficits observed in the FASDs. Although our study was focused on FASD, low fetal folate may also be part of the aetiologies of diseases seen in children born to chronic alcohol drinking women during pregnancy. Although future studies are needed to address the mechanism for the decreased folate transfer, current practice should continue to properly counsel pregnant women and women of childbearing age on proper folic acid supplementation as well as abstinence from alcohol during pregnancy.

## References

[1] Giugliani ER, Jorge SM, Goncalves AL (1985). Serum and red blood cell folate levels in parturients, in the intervillous space of the placenta and in full-term newborns.. J Perinat Med.

[2] Economides DL, Ferguson J, Mackenzie IZ, Darley J, Ware II (1992). Folate and vitamin B12 concentrations in maternal and fetal blood, and amniotic fluid in second trimester pregnancies complicated by neural tube defects.. Br J Obstet Gynaecol.

[3] Guerra-Shinohara EM, Morita OE, Peres S, Pagliusi RA, Sampaio Neto LF (2004). Low ratio of S-adenosylmethionine to S-adenosylhomocysteine is associated with vitamin deficiency in Brazilian pregnant women and newborns.. Am J Clin Nutr.

[4] Molloy AM, Mills JL, Cox C, Daly SF, Conley M (2005). Choline and homocysteine interrelations in umbilical cord and maternal plasma at delivery.. Am J Clin Nutr.

[5] Navarro J, Causse MB, Desquilbet N, Herve F, Lallemand D (1984). The vitamin status of low birth weight infants and their mothers.. J Pediatr Gastroenterol Nutr.

[6] Obeid R, Munz W, Jager M, Schmidt W, Herrmann W (2005). Biochemical indexes of the B vitamins in cord serum are predicted by maternal B vitamin status.. Am J Clin Nutr.

[7] Relton CL, Pearce MS, Parker L (2005). The influence of erythrocyte folate and serum vitamin B12 status on birth weight.. Br J Nutr.

[8] Stark KD, Pawlosky RJ, Sokol RJ, Hannigan JH, Salem NJ (2007). Maternal smoking is associated with decreased 5-methyltetrahydrofolate in cord plasma.. Am J Clin Nutr.

[9] Thorand B, Pietrzik K, Prinz-Langenohl R, Hages M, Holzgreve W (1996). Maternal and fetal serum and red blood cell folate and vitamin B12 concentrations in pregnancies affected by neural tube defects.. Z Geburtshilfe Neonatol.

[10] Wallace JM, Bonham MP, Strain J, Duffy EM, Robson PJ (2008). Homocysteine concentration, related B vitamins, and betaine in pregnant women recruited to the Seychelles Child Development Study.. Am J Clin Nutr.

[11] Henderson GI, Perez T, Schenker S, Mackins J, Antony AC (1995). Maternal-to-fetal transfer of 5-methyltetrahydrofolate by the perfused human placental cotyledon: evidence for a concentrative role by placental folate receptors in fetal folate delivery.. J Lab Clin Med.

[12] Bisseling TM, Steegers EA, van den Heuvel JJ, Siero HL, van de Water FM (2004). Placental folate transport and binding are not impaired in pregnancies complicated by fetal growth restriction.. Placenta.

[13] Solanky N, Requena Jimenez A, D'Souza SW, Sibley CP, Glazier JD (2010). Expression of folate transporters in human placenta and implications for homocysteine metabolism.. Placenta.

[14] Keating E, Goncalves P, Campos I, Costa F, Martel F (2009). Folic acid uptake by the human syncytiotrophoblast: interference by pharmacotherapy, drugs of abuse and pathological conditions.. Reprod Toxicol.

[15] Prasad PD, Ramamoorthy S, Leibach FH, Ganapathy V (1995). Molecular cloning of the human placental folate transporter.. Biochem Biophys Res Commun.

[16] Yasuda S, Hasui S, Kobayashi M, Itagaki S, Hirano T (2008). The mechanism of carrier-mediated transport of folates in BeWo cells: the involvement of heme carrier protein 1 in placental folate transport.. Biosci Biotechnol Biochem.

[17] Fisher SE, Inselman LS, Duffy L, Atkinson M, Spencer H (1985). Ethanol and fetal nutrition: effect of chronic ethanol exposure on rat placental growth and membrane-associated folic acid receptor binding activity.. J Pediatr Gastroenterol Nutr.

[18] Keating E, Lemos C, Goncalves P, Martel F (2008). Acute and chronic effects of some dietary bioactive compounds on folic acid uptake and on the expression of folic acid transporters by the human trophoblast cell line BeWo.. J Nutr Biochem.

[19] Goh YI, Koren G (2008). Folic acid in pregnancy and fetal outcomes.. J Obstet Gynaecol.

[20] Chudley AE, Conry J, Cook JL, Loock C, Rosales T (2005). Fetal alcohol spectrum disorder: Canadian guidelines for diagnosis.. CMAJ.

[21] Jones KL (2006). Smith's recognizable patterns of human malformation..

[22] Schlotz W, Jones A, Phillips DI, Gale CR, Robinson SM (2010). Lower maternal folate status in early pregnancy is associated with childhood hyperactivity and peer problems in offspring.. J Child Psychol Psychiatry.

[23] Veena SR, Krishnaveni GV, Srinivasan K, Wills AK, Muthayya S (2010). Higher maternal plasma folate but not vitamin B-12 concentrations during pregnancy are associated with better cognitive function scores in 9- to 10- year-old children in South India.. J Nutr.

[24] Gundogan F, Elwood G, Mark P, Feijoo A, Longato L (2010). Ethanol-Induced Oxidative Stress and Mitochondrial Dysfunction in Rat Placenta: Relevance to Pregnancy Loss.. Alcohol Clin Exp Res.

[25] Cano MJ, Ayala A, Murillo ML, Carreras O (2001). Protective effect of folic acid against oxidative stress produced in 21-day postpartum rats by maternal-ethanol chronic consumption during pregnancy and lactation period.. Free Radic Res.

[26] Kapur BM, Khuu M, Bennett D, Tran S, Lehotay D (2009). Endogenous methanol derived formic acid correlates with cognitive dysfunction in children born to drinking mothers.. Alcohol Clin Exp Res.

[27] Kapur BM, Vandenbroucke AC, Adamchik Y, Lehotay DC, Carlen PL (2007). Formic acid, a novel metabolite of chronic ethanol abuse, causes neurotoxicity, which is prevented by folic acid.. Alcohol Clin Exp Res.

[28] Sokoro AA, Zhang Z, Eichhorst JC, Zello GA, House JD (2008). Formate pharmacokinetics during formate administration in folate-deficient young swine.. Metabolism.

[29] Lin GW (1991). Maternal-fetal folate transfer: effect of ethanol and dietary folate deficiency.. Alcohol.

[30] Gutierrez CM, Ribeiro CN, de Lima GA, Yanaguita MY, Peres LC (2007). An experimental study on the effects of ethanol and folic acid deficiency, alone or in combination, on pregnant Swiss mice.. Pathology.

[31] Halsted CH, Villanueva JA, Devlin AM, Chandler CJ (2002). Metabolic interactions of alcohol and folate.. J Nutr.

[32] Larroque B, Kaminski M, Lelong N, d'Herbomez M, Dehaene P (1992). Folate status during pregnancy: relationship with alcohol consumption, other maternal risk factors and pregnancy outcome.. Eur J Obstet Gynecol Reprod Biol.

[33] Stark KD, Pawlosky RJ, Beblo S, Murthy M, Flanagan VP (2005). Status of plasma folate after folic acid fortification of the food supply in pregnant African American women and the influences of diet, smoking, and alcohol consumption.. Am J Clin Nutr.

[34] Lin GW-J (1988). Folate deficiency and acute ethanol treatment on pregnancy outcome in the rat.. Nutrition Research.

[35] Wang LL, Zhang Z, Li Q, Yang R, Pei X (2009). Ethanol exposure induces differential microRNA and target gene expression and teratogenic effects which can be suppressed by folic acid supplementation.. Hum Reprod.

[36] Xu Y, Tang Y, Li Y (2008). Effect of folic acid on prenatal alcohol-induced modification of brain proteome in mice.. Br J Nutr.

[37] Yanaguita MY, Gutierrez CM, Ribeiro CN, Lima GA, Machado HR (2008). Pregnancy outcome in ethanol-treated mice with folic acid supplementation in saccharose.. Childs Nerv Syst.

[38] Garcia-Rodriguez S, Arguelles S, Llopis R, Murillo ML, Machado A (2003). Effect of prenatal exposure to ethanol on hepatic elongation factor-2 and proteome in 21 d old rats: protective effect of folic acid.. Free Radic Biol Med.

[39] Wentzel P, Eriksson UJ (2008). Folic acid prevention of ethanol damage in rat offspring.. Alcohol Clin Exp Res.

[40] Antony AC (2007). In utero physiology: role of folic acid in nutrient delivery and fetal development.. Am J Clin Nutr.

[41] Kim JM, Hong K, Lee JH, Lee S, Chang N (2009). Effect of folate deficiency on placental DNA methylation in hyperhomocysteinemic rats.. J Nutr Biochem.

[42] Gallou-Kabani C, Gabory A, Tost J, Karimi M, Mayeur S (2010). Sex- and diet-specific changes of imprinted gene expression and DNA methylation in mouse placenta under a high-fat diet.. PLoS ONE.

[43] Axelrod J, Daly J (1965). Pituitary gland: enzymic formation of methanol from S-adenosylmethionine.. Science.

